# Mr. Ring, on the Use of Spongia Usta

**Published:** 1804-07-01

**Authors:** John Ring

**Affiliations:** New Street, Hanover Square


### *To the Editors of the Medical and Phyſical Journal.*

GENTLEMEN,

I Have sent the Resolution of the Grand Jury at the Assizes at Horsham, relative to the extermination of the small-pox from the county of Sussex; an example which I hope, will soon be followed by every county in England.

In

In consequence of this resolution, I have the pleasure to inform you, a society is already formed; of which His Royal Highness the Prince of Wales is Patron, and Lord Pelham President. It is also supported by the Members for the county, and many other persons of distinction.

I hope soon to receive a copy of the plan of the Society, which I shall not fail to transmit.

I have lately received letters from Dr. M'Dermot, of Coolavin, near Boyle, in Ireland; informing me, that he has experienced the efficacy of the spongia usta in the shape of lozenges, which he never was so fortunate as to experience, when it was given in any other way. He speaks in very handsome terms of your Journal, which was the channel through which I conveyed to the public my remarks on the subject; from a perusal of which, Dr. M'Dermot was induced to try the practice. He has one obstinate case under his care, which has resisted this and every other remedy that is commonly employed for the disease.

Since I published my observations on the subject, as well as before that time, I have met with some cases which I could not cure; but a greater number which have yielded to the medicine before-mentioned; among which was one of above forty years standing, which greatly impeded respiration and deglutition, and rendered the life of the woman who laboured under it perfectly miserable.

Although the burnt sponge certainly is more efficacious in the form of a lozenge, yet, in one instance, where the patient could not take it in that manner, it succeeded very readily in the form of pills. This case is the only one in which I have known the complaint yield readily, when the medicine was swallowed at once; though I have often given it in pills, as well as in an electuary, and in a liquid form.

How far the bronchocele partakes of the nature of scrofula has been made a question; debility appears to be the pre-disposing cause of both these affections, which occur much more commonly in those of a relaxed habit than in others; and much more commonly in women than in men. I am, &c.

JOHN RING.

*New Street, Hanover Square,*
*June* 14, 1804.

*Resolutions*

